# The Impact of the COVID-19 Pandemic on the Organization of Cardio-Hematology Care—A Polish Single Center Experience

**DOI:** 10.3390/medicina58030337

**Published:** 2022-02-23

**Authors:** Piotr Gościniak, Bartłomiej Baumert, Sławomir Milczarek, Joanna Jędrzychowska-Baraniak, Anna Sobuś, Bogusław Machaliński

**Affiliations:** 1 Laboratory of Non-Invasive Cardiac Imaging, Independent Public Clinical Hospital Nr 1, Pomeranian Medical University, 71-252 Szczecin, Poland; piotrgosciniak1@gmail.com (P.G.); joanna.baraniak@wp.pl (J.J.-B.); 2 Department of Endocrinology, Metabolic and Internal Diseases, Pomeranian Medical University, 71-252 Szczecin, Poland; 3 Department of General Pathology, Pomeranian Medical University, 70-111 Szczecin, Poland; bbaumert@pum.edu.pl (B.B.); slawek.milczarek@gmail.com (S.M.); ania.sobus@gmail.com (A.S.); 4 Department of Hematology and Transplantology, Pomeranian Medical University, 71-252 Szczecin, Poland

**Keywords:** blood diseases, cardiac complications, cardio-hematology care, chemotherapy, COVID-19 pandemic

## Abstract

*Background and Objectives*: We present a retrospective report on the cardio-hematological care of hematology patients at a university hospital in Poland during the COVID-19 pandemic. *Materials and Methods*: The number of hospitalizations at the Hematology Department and cardio-hematology consultations throughout 2019 and 2020 was analyzed. The types of cardiac procedures, risk factors, and complications were also assessed. *Results*: A significant reduction in the number of hospitalizations was observed in 2020 as compared to 2019. However, there were no significant differences in the incidence of hematological diseases between both of the analyzed years. In 2019, 299 cardiac consultations were performed in hematological patients, and there was a total of 352 such consultations performed in 2020 (*p* = 0.042). Less high-risk tests (transesophageal and stress echocardiography) were performed in 2020, in favor of the use of cardiac computed tomography in cardiac diagnostics as it was safer during the pandemic. At least one cardiovascular risk factor during cardiac consultation was noted in 42% and 48% of hematological patients in 2019 and 2020, respectively. Among 651 examined hematological patients, the most common findings were mild cardiac complications of hemato-oncological treatment, which were found in 57 patients. *Conclusions*: This study seems to confirm that during a pandemic there is an increased demand for well-organized cardio-hematology consultations.

## 1. Introduction

Hematological patients have a high risk of a severe COVID-19 infection and are prone to cardiac complications due to immunodeficiency and applied chemotherapy. There are no data on the care of hematology patients in Poland during the pandemic. Blood cancers in Poland account for approximately 4% of oncology cases, which corresponds to about 7500 new cases annually. Before the COVID-19 pandemic, in the West Pomeranian Province, Poland, around 3000 hospitalizations for neoplasms of the hematopoietic and lymphatic systems in adults were recorded, and mature B-cell neoplasms resulted in 1685 hospitalizations annually. There were 199 hospitalizations per 100,000 adults in the West Pomeranian Province, the second highest rate in the country [1].

Chemotherapy remains the main treatment method in hematology. Treatment regimens include the use of anthracyclines, vascular endothelial growth factor inhibitors (VEGF-I), Bcr-Abl gene kinase inhibitors, 5-fluorouracil and its metabolites, proteasome inhibitors, and tyrosine kinase inhibitors. All these medicines have a cardiotoxic effect and can damage the heart muscle through a specific mechanism (mostly by inducing heart failure, arrhythmia, or myocardial infarction). As well as classic anthracyclines, new molecularly targeted medicines have also been shown to influence cell pathways, damaging cardiomyocytes. For this reason, cardiac monitoring of patients is necessary before, during, and after hematological treatment [2]. Unfortunately, only some hematology centers in Poland offer permanent cardiology care to their patients.

In 2020, in the face of the rapidly growing COVID-19 pandemic in north-west Poland, there was an urgent need to reorganize health care facilities to ensure the safety and adequate protection of those infected, those suspected of being infected, other patients, and health care workers. 

After the lockdown was announced, many scheduled visits to primary care physicians and specialists, including hematologists and cardiologists, were suspended, often on the decision of the patients themselves, for fear of infection. This resulted in significant diagnostic delays in numerous groups of patients [3]. Early diagnosis and rapid treatment is associated with optimal treatment results, particularly for hematological and oncological patients. Currently, there is a concern, not only in Poland, that the number of cancer deaths will increase as a result of the diagnostic delays caused by the COVID-19 pandemic. For example, it is estimated that in patients with colorectal cancer, the number of deaths may increase by up to 15% [4]. Similar problems were observed in cardiology, where the number of diagnoses of acute coronary syndromes decreased by over 40% in some regions of Poland [5]. The same observations from Turkey indicate a 47.1% decrease in acute myocardial infarction (AMI) admissions during the pandemic [6]. The number of Polish patients with decompensated heart failure also increased due to the lack of adequate access to health care [7].

There are still only a few publications available on cardio-hematology-specific care [8]. The guidelines and recommendations of scientific societies emphasize that delaying or avoiding cardiology care in the COVID-19 pandemic may result in an increased rate of adverse events in patients undergoing chemotherapy [9]. Therefore, with each scheduled cardiological consultation, a careful risk-benefit assessment should be performed and the recommendations should be followed [10]. 

The occurrence of COVID-19 in hematological patients was also assessed. It has been shown that the incidence of SARS-CoV-2 infection in patients with hematological cancers was not significantly higher than in the general population [11]. However, patients receiving anti-cancer therapy during COVID-19 frequently discontinued treatment. 

Another meta-analysis showed that 16.5% of cancer patients with confirmed SARS-CoV-2 infection were patients with hematological malignancies [12].

Hematological patients presented a 3–4 times higher rate of severe course and mortality when compared to infected patients from the general population. In particular, higher mortality rates were found in patients with AML (44%) and myelodysplastic syndrome (42%). In addition, the type of chemotherapy appeared to be associated with mortality [13]. 

COVID-19 cardiac symptoms may include myocarditis, myocardial infarction, heart failure, acute coronary syndrome, and arrhythmia. Various signaling pathways could be altered in the infected cardiomyocytes, causing cardiac dysfunction as heart tissue shows high levels of necessary accessory proteins such as ACE2, NRP-1, TMPRSS2, CD147, integrin α5β1, and CTSB/L, which are targets of the SARS-CoV-2 virus [14].

The objective of the study was to estimate the impact of the COVID-19 pandemic on the cardio-hematology care of patients at a university hospital with a hematology referral center.

## 2. Materials and Methods

The observational registry data for this study were obtained from standard medical documents and there was no effect on patient treatment; therefore, the approval of the ethics committee was not required. The observational data for the years 2019 and 2020 were collected from the large Independent Public Clinical Hospital (IPCH), the Laboratory for Non-Invasive Cardiac Diagnostics and Cardio-Oncology at IPCH, as well as from the Hematology Clinic, which is the referral department in the West Pomeranian Province, with one of the highest number of hospitalizations in Poland. It consists of the Department of Hematology, the Department of Bone Marrow Transplantation, and the Chemotherapy Outpatient Department. Both classical cytostatic drugs and immunochemotherapy are used in the treatment of blood cancers in the Hematology Clinic. The IPCH has a predominant oncological profile. The hospital does not, however, have a cardiology department.

In the Laboratory for Non-Invasive Cardiac Diagnostics at IPCH, accredited by the Echocardiography Section of the Polish Cardiac Society, all types of echocardiography (transthoracic 2D and 3D, transesophageal 2D and 3D, dobutamine stress, contrast, and transcranial) were performed using the GE VIVID 6 and Philips Epiq CVx3D systems. Additionally, there was access to cardiac and coronary tomography as well as SPECT CTs of the heart. 

In order to improve operational organization, the working time of the laboratory was extended. This was necessary to properly prepare for each consultation (i.e., disinfection and epidemiological interview with the next patient). FFP2 and FFP3 masks were worn. Cardiological consultations were adjusted to only occur during the patient’s stay in the Department of Hematology. The schedule of the consultation and chemotherapy administration was mutually agreed upon for each appointment. All patients were informed at their first visit about possible cardiac complications and they were advised to personally report any cardiac dysfunction. 

During consultations at the Laboratory for Non-Invasive Cardiac Diagnostics, each patient had a physical examination, a resting electrocardiography (ECG), a resting echocardiography, and when the quality of imaging made it technically feasible, an assessment of the global systolic longitudinal deformation of the left and right ventricular myocardium (GLS, TOMTEC, Philips, Amsterdam, The Netherlands), as well as ejection fraction (EF) using automatic artificial intelligence algorithms in three dimensions (Dynamic Heart Model, Philips). More advanced tests were performed depending on any indications. Usually, visits were planned before the start of chemotherapy, six months later, and then after the end of treatment. A cardiac follow up was recommended for all patients receiving cardio-hematology consultations. 

However, some hematological patients were considered at a high risk of cardiovascular complications: those who had received a high dose of anthracyclines in the past, a high dose of radiotherapy in the area of the heart, simultaneous treatment with anthracycline and radiotherapy, those who experienced a vascular incident, those who had two risk factors for cardiovascular events or had impaired left ventricular ejection fraction, had a current heart disease (valvular heart disease, cardiomyopathy), and those over 60 years of age [15]. In this particular group, a supplementary visit was planned after the first month of treatment. All follow-up examinations took place in person. Only hospitalized patients were assessed. All patients admitted to the hospital were tested for SARS-CoV-2. A positive result postponed the admission. Therefore, only incidental consultations were performed in SARS-CoV-2 positive patients. Telehealth was considered inadequate in this particular population due to specific hematological treatments (usually consisting of anthracyclines or other potentially highly cardiotoxic drugs). 

Statistical analyses were performed in SAS ver. 9.4 (SAS Inc., Cary, NC, USA). Continuous variables are reported as mean ± SD and differences were compared using a Student’s *t*-test. Categorical variables are expressed as counts and percentages. The numbers of consultations were treated as events and the differences were compared by the chi2 test of independence, the chi2 test for equal proportion or the Fisher exact test, as appropriate. The number of consultations in an individual month was treated as a continuous variable and the paired signed-rank test (Wilcoxon) compared the distributions for 2019 and 2020. The p-values were two-sided and the significance level was set at 0.05.

## 3. Results

### 3.1. Hospitalizations at the Hematology Clinic before and during the Pandemic

**Figure 1:**
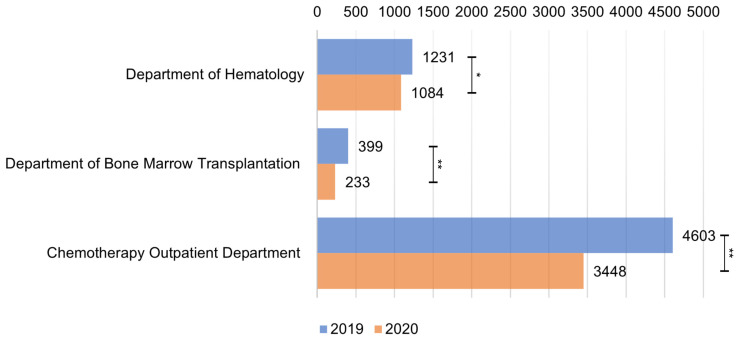
Comparison of the number of hospitalizations between 2019 and 2020 in each of the individual departments of the Hematology Clinic. Significance levels are indicated by asterisks: * = *p* < 0.01 and ** = *p* < 0.001.

**Figure 2:**
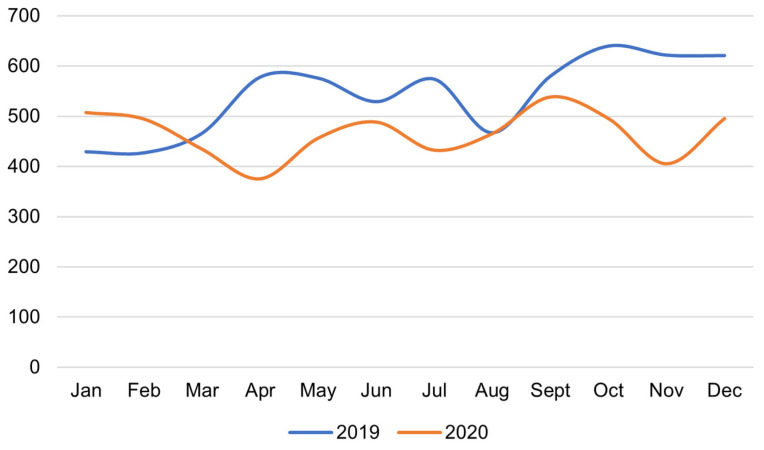
Comparison of the number of hospitalizations between individual months in the Hematology Clinic during 2019 and 2020.

The total number of hospitalizations at the Hematology Clinic was 6233 in 2019 and decreased to 4765 in 2020 (*p* < 0.001) (Figure 1). Differences are also noted between individual months (Figure 2). 

Indolent lymphoproliferation was the most common reason for hospitalization in the Hematology Clinic in both 2019 and 2020, followed by aggressive lymphoma, acute myeloid leukemia, lymphoblastic leukemia, and multiple myeloma. 

### 3.2. Cardiology Consultations

**Figure 3:**
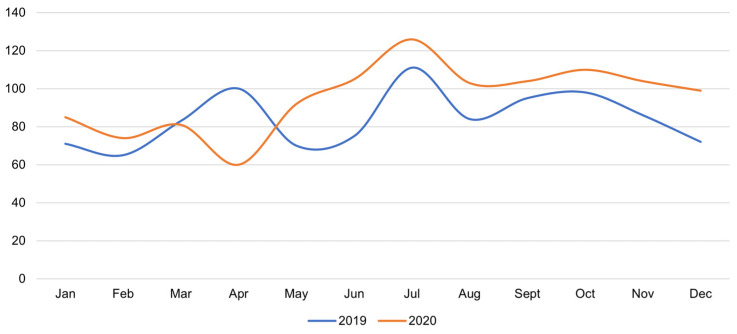
Comparison of the number of inpatient cardiology consultations between 2019 (total *n* = 1010) and 2020 (total *n* = 1143) at IPCH. The *p*-values for 2019 vs. 2020 are *p* = 0.0042 (using the chi2 test for equal proportion for totals of 1010 and 1143) and *p* = 0.040 (using the Wilcoxon rank test comparing the 2019 and 2020 distributions).

The number of inpatient cardiology consultations at IPCH in 2019 and throughout 2020 was analyzed, although the pandemic was not announced until the first two months of 2020. Only hospitalized patients were assessed. In total, 1010 inpatient cardiology consultations were performed at IPCH in 2019 and 1143 in 2020, including 92 people infected with COVID-19. Figure 3 shows consultations performed in the Laboratory for Non-Invasive Cardiac Diagnostics and Cardio-Oncology at IPCH for inpatient patients from all Departments of IPCH. Table 1 shows the types and number of cardiac procedures performed for inpatients receiving cardiology consultations during 2019 and 2020.

**Figure 4:**
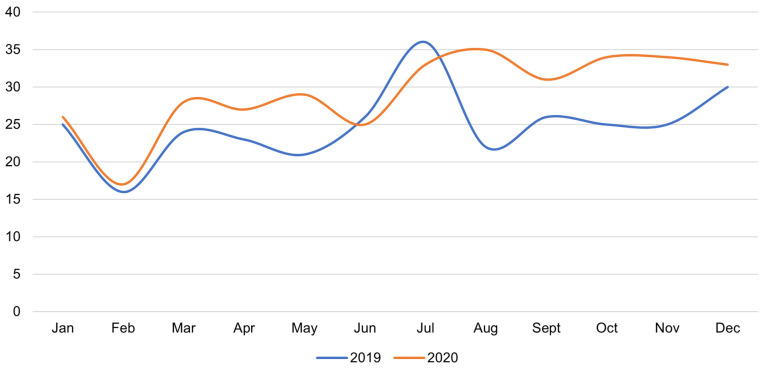
Comparison of the number of inpatient cardiac consultations from the Hematology Clinic for each month at the Laboratory for Non-Invasive Cardiac Diagnostics (2019: *n* = 299; 2020: *n* = 352), *p* = 0.042.

Regarding the Hematology Clinic, in 2019, 299 cardiac consultations were performed, and there was a total of 352 such consultations in 2020 (Figure 4). The general characteristics of hematological patients receiving cardiology consultations are presented in Table 2. At least one cardiovascular risk factor was noted during cardiac consultation in as many as 126 (42%) and 169 (48%) of patients in 2019 and 2020, respectively.

A new cardiac incident at any follow-up visit was considered to be a cardiac complication. In order of their frequency of occurrence, complications included uncontrolled arterial hypertension, arrhythmias and conduction disorders, exacerbation of coronary artery disease, thromboembolism, and heart failure with a reduced ejection fraction, which was reported particularly rarely (Table 3). Cardiac events due to hemato-oncological therapy were observed in 27 and 30 patients in 2019 and 2020, respectively. There were no significant differences in the incidence of cardiac complications between 2019 and 2020. Likewise, some of the patients had more than one complication, e.g., arterial hypertension and arrhythmia.

## 4. Discussion

Regardless of the type of Hematology Clinic department, a significant reduction in the number of hospitalizations was observed in 2020 when compared to the previous year. In 2019, the distribution of patients in individual months was stable. In 2020, after the initial shock of the complete lockdown, the number of patients grew steadily from May to July, and then remained constant.

Despite the decrease in the number of hospitalizations at IPCH, the number of inpatient cardiology consultations performed in 2020 increased. The number of transthoracic echocardiographic examinations also went up. This was due to a higher number of transthoracic echocardiographic examinations in 2020, whereas the use of dobutamine stress and transesophageal echocardiography declined. Significantly more cardiac examinations were performed using computed tomography.

The number of cardio-hematology consultations increased in 2020 (*n* = 352) compared to 2019 (*n* = 299), even though the pandemic did not change the cardiac monitoring protocol in all of these patients. The compared groups of blood cancer patients who had cardiac examinations in 2019 and 2020 had a similar age and gender distribution. The presence of risk factors for cardiotoxicity, other than coronary artery disease, did not show significant differences in the studied populations. At least one cardiovascular risk factor was noted in as many as 126 (42%) and 169 (48%) patients in 2019 and 2020, respectively. There were no significant differences in the incidence of hematological diseases in both years analyzed.

The introduction of the lockdown and the slogan “stay home, save lives, and protect the health service”, out of fear for a complete collapse of the health care system, could have contributed to a decrease in the number of all hospitalizations, diagnostic tests, hematological hospitalizations, and inpatient consultations at the Laboratory for Non-Invasive Cardiac Diagnostics during the first months of the pandemic in 2020.

Initially, the overall decrease in the number of hospitalizations and scheduled visits to our center was likely due to the fear of the spread of the new disease and the urgent need to adapt the hospital and wards to new procedures during the pandemic. This led to the reduction or postponement of scheduled diagnostics, or even the cancellation of some appointments in the first half of 2020. Moreover, disruptions in public transport may have hampered access to specialized diagnostic procedures. Another drop in hospitalizations in the late fall was likely related to the second wave of the disease, which affected both patients and hospital staff.

As a consequence, patients with blood cancers unexpectedly suffered from delays in diagnostics and therapies all over the world [16]. In order to properly treat these patients without reducing the chances of curing them, numerous national, European, and American guidelines for the management of patients during the COVID-19 pandemic [17] were developed relatively quickly and updated on an ongoing basis. Most scientific societies changed their treatment and control schedules slightly, depending on the severity of the disease. Chemotherapy protocols were updated to reduce the frequency of appointments and minimize the incidence of high immunosuppression [18]. It was recommended, inter alia, that treatment strategies, such as allogeneic or autologous stem cell transplantation, be delayed [17], as this group of patients was shown to have higher mortality rates from COVID-19 [19].

Despite the pandemic and the recommendations of epidemiological scientific societies, including the International Cardio-Oncology Society (ICOS) in 2020 [20], to reduce the frequency and scope of close contact imaging tests, such as echocardiography, in our laboratory during each consultation patients had ECGs and echocardiography, as well as immediate additional tests (e.g., dobutamine and transesophageal) if necessary. Although, before being admitted to the hospital, all patients had COVID-19 swab tests (RT-PCR), sometimes it was also necessary to examine infected patients. In such cases, the recommendations of the Echocardiography Section of the Polish Cardiac Society were followed [21].

The compared groups of hematological patients receiving cardiology consultations proved to be quite homogeneous. We observed slightly fewer risk factors for cardiovascular events as compared to those reported in the literature, which may be due to the relatively younger age of the studied patients [22,23]. In the 651 consulted patients, cardiac complications due to hemato-oncological therapy were identified in 57 patients, of which most were mild. All received early intervention since timely diagnosis can prevent or shorten interruptions in access to chemotherapy [24].

The COVID-19 pandemic had a large impact on hospitalizations and the use of chemotherapy. The increase in the number of cardio-hematology consultations could have resulted from hematologists having a greater awareness of the need for cardiac assessment of qualifying patients and patients undergoing anti-cancer therapy, as well as the knowledge possessed by cardiologists regarding the need for special monitoring of such patients. These deliberate and collaborative efforts made by cardiologists and hematologists led to the development of management protocols to optimize the cardiovascular health of patients and minimize interruptions in the use of anti-cancer therapy associated with the treatment of heart diseases.

## 5. Study Limitations

This study presents data from only one university center in northwest Poland; however, it concerns the largest hospital complex in the West Pomeranian Province, although in other parts of Poland, even more COVID-19 infections have occurred. Cardiac complications only concerned patients who came to the follow-up visit at our center and therefore may be underestimated if they happened at the other regional center. Detailed echocardiographic data obtained will be analyzed separately and published elsewhere.

## 6. Conclusions

1. During the COVID-19 pandemic, there was a significant decrease in the total number of hospitalizations, diagnostic tests, and chemotherapy in the hematology referral center.
2. During the pandemic, there was an increase in the number of cardio-hematology consultations.
3. Despite the pandemic, a wide range of diagnostic tests were performed, although less high-risk (transesophageal and stress) tests were performed in accordance with the recommendations of the European and Polish imaging societies.
4. In accordance with the recommendations of scientific societies, there was a significant increase in the use of cardiac computed tomography in cardiac diagnostics as it was safer during the pandemic.

## Figures and Tables

**Table 1:** Comparison of the number of procedures performed for inpatients receiving cardiology consultations from all departments of IPCH during 2019 and 2020.

Type of Cardiac Procedure	№ of Procedures in 2019	№ of Procedures in 2020	*p*
Transthoracic echocardiography	878	906	0.434
Transesophageal echocardiography	32	4	<0.001
Dobutamine echocardiography	114	28	<0.001
Transcranial doppler echocardiography	44	48	0.677
Cardiac tomography	14	107	<0.001
SPECT CT	19	17	0.739
COVID-19+ echocardiography	-	92	-

**Table 2:** General characteristics of hematological patients receiving consultations at the Laboratory for Non-Invasive Cardiac Diagnostics in 2019 and 2020.

Characteristics of Consulted Patients		2019	2020	*p*
Number of consultations		*n* = 299	*n* = 352	0.038
Age (mean ± SD)		61 ± 14	61 ± 14	0.980
Women (%)		40	44	0.315
Risk factors before chemotherapy (%)				
Hypertension		20	20	1.0
Coronary artery disease		19	9	<0.001
Valvular heart disease		3	4	0.505
Arrhythmias		6.5	9	0.253
Pulmonary embolism		3	3	1.00
Diabetes		6	5	0.542
Blood diseases (%)				
Acute lymphocytic leukemia		7	5	0.306
Acute myeloid leukemia		28	28	1.00
Lymphomas	aggressive B cell	34	31	0.392
	indolent B cell	15	23	0.010
	T cell	3	4	0.505
Multiple myeloma		10.5	6	0.039

**Table 3:** New cardiac complications found during the follow-up of hemato-oncological patients (2019: *n* = 27; 2020: *n* = 30; *p* = 0.211).

New Cardiac Complications	2019 (*n* = 27)	2020 (*n* = 30)
Poorly controlled arterial hypertension	6	9
Paroxysmal supraventricular tachycardia	4	7
Atrial fibrillation/atrial flutter	3	2
Premature ventricular contractions	6	3
Conduction disorders	1	1
Exacerbation of coronary artery disease	2	4
Acute coronary syndrome	0	2
Thromboembolism	3	2
Systolic heart failure	2	0

## Data Availability

Data available upon request from B.M.

## Abbreviations

ACS	acute coronary syndrome
AF	atrial fibrillation
AMI	acute myocardial infarction
AT	atrial flutter
CAD	coronary artery disease
ECG	electrocardiography
EF	ejection fraction
HF	heart failure
PSVT	paroxysmal supraventricular tachycardia
IPCH	Independent Public Clinical Hospital
PVC	premature ventricular contractions
SPECT CT	single photon emission computed tomography
VEGF-I	vascular endothelial growth factor inhibitors
